# Behavioural Constraints to Home Range Allometries in Aquatic Organisms

**DOI:** 10.1002/ece3.71886

**Published:** 2025-07-30

**Authors:** Vanessa Marrocco, Milad Shokri, Flavio Monti, Francesco Cozzoli, Fabio Vignes, Alberto Basset

**Affiliations:** ^1^ LifeWatch ERIC, Service Centre Lecce Italy; ^2^ Laboratory of Ecology, Department of Biological and Environmental Sciences and Technologies University of Salento Lecce Italy; ^3^ National Biodiversity Future Centre (NBFC) Palermo Italy; ^4^ Institute of Research on Terrestrial Ecosystems (IRET), National Research Council (CNR) Lecce Italy

**Keywords:** allometry, aquatic organisms, body size, home range, space use behaviour

## Abstract

Home range is the spatial evolutionary arena where an individual can succeed or fail. Its size reflects the balance between individual energy requirements and acquisitions. Since various behavioural traits can affect individual energy demand, we investigated how these traits affect individual space‐use behavior and resulting home range size of aquatic organisms. We compiled data for 172 freshwater and marine species, ranging from invertebrates to marine mammals, spanning 8 orders of magnitude in body size and 13 in home range size. Across all taxa, home range size scaled allometrically with body size (*R* ^2^ = 0.78, exponent = 1.68 ± 0.07). Behavioural traits explained an additional 27.15% of the variance beyond that explained by body size alone. In particular, carnivorous and pelagic species exhibited significantly larger home ranges than herbivorous and benthic ones, whereas migration and sociality were not retained as significant predictors beyond body size, despite migration affecting the intercept of the scaling relationship. We show that while body size is a strong predictor of home range, behavioural categories significantly modulate the allometric relationship. Our findings provide new insights into the behavioural constraints shaping spatial ecology in aquatic environments. These results underscore the role of energy‐demanding behaviours in shaping spatial ecology, with implications for individual density, the intensity of intra‐specific interactions, and patterns of interspecific coexistence.

## Graphical abstract

We conducted a meta‐analysis on the scaling of home range size with body size across 172 species, from invertebrates to marine mammals. Our findings revealed that behavioural traits significantly influence this scaling. Specifically, trophic and pelagic behaviours, which extend beyond the influences of body size, significantly impacted home range sizes. In contrast, social and migratory behaviours had a lesser effect on variations in home range size.

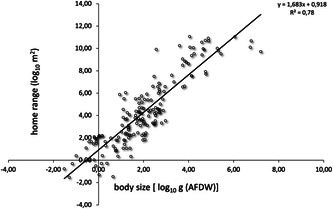

## 1 Introduction

Home range is the spatial expression of all behaviours that an individual performs to survive and reproduce (Burt 1943). The ratio of resource productivity to individual energy requirement has been proposed as a conservative quantification of the individual home range size (Harestad and Bunnel 1979). Assuming that space use behaviour is the outcome of decision‐making processes shaped by natural selection, these processes aim to optimise the contribution of spatially distributed resources to individual fitness (Mitchell and Powell 2004). Following this, a home range represents an interplay between the environment and the animal's perception of resource availability and profitability (Powell 2000; Börger et al. 2008), acting as a spatial evolutionary arena, both at the intra‐ (Jetz et al. 2004) and interspecific levels (Basset 1995).

Despite numerous seminal studies in this field, the mechanistic understanding of the criteria underlying the setting of individual home range position, surface area, dynamics, and spatial variations still needs further exploration. Disentangling these variations is crucial, as they significantly influence ecological processes and community organisation, including species interactions and mechanisms of coexistence (e.g., Wang and Grimm 2007; Börger et al. 2008; Kotler et al. 1993).

Animals use space for a variety of purposes, navigating trade‐offs with their daily and lifetime requirements (e.g., van Beest et al. 2011). They employ various strategies to harvest energy in accordance with the principle of space and resource use optimisation (MacArthur and Pianka 1966; Kotler et al. 1993), while simultaneously balancing net energy gain against predation and other requirements for survival and reproduction (e.g., Brown and Kotler 2004). Home range size is critically influenced by an animal's body size, which dictates its maintenance energy requirements, as these requirements increase with size (Brown et al. 2004; McNab 1963). Larger animals, with their higher energy demands, typically require a larger space, either bi‐ or tri‐dimensional, to secure sufficient resources to sustain their energy needs, whereas smaller ones can manage within more confined spaces (Basset 1995).

However, the relationship between home range size and body size, along with its scaling exponents, remains a subject of debate and active research (e.g., Makarieva et al. 2004; Haskell et al. 2002; Byrnes et al. 2023; Shokri et al. 2025). Several studies have identified an allometric relationship between home range size and body size, with scaling exponents similar to those observed in metabolic scaling, which typically range from 2/3 to 3/4 (West et al. 1997; Peters 1983). Conversely, other studies suggest a scaling exponent that is steeper than the typical power law, indicating hyper‐allometric scaling (Harestad and Bunnel 1979; Lindstedt et al. 1986).

The observed variations in home range sizes across different species can be attributed to a complex interplay of multiple ecological and biological factors. For example, the foraging behaviour and the fractal distribution of resources may alter the size scaling of home range (Haskell et al. 2002; Edelsparre et al. 2021). This spatial distribution necessitates adaptations of different sized conspecifics to efficiently exploit available resources. The trophic diet and its breadth, which can vary also among individuals of a species, can further alter the relationship between individual body size and home range size. This can be particularly highlighted in predatory species, where the availability of prey can expand or limit the necessary roaming area (Makarieva et al. 2005).

On the other hand, thermoregulation strategies might influence the spatial needs of different species as they adjust their ranges to maintain optimal body temperatures (Hendriks 2007). Similarly, movement rates and locomotion strategies could substantially influence the extent of space use, as animals with different mobility patterns—such as benthic vs. pelagic lifestyles—require varying distances to access sufficient resources (Tamburello et al. 2015). Additionally, these spatial requirements can change during different life stages or ontogeny, introducing further variations in the relationship between body size and home range size (Haskell et al. 2002; Bejan and Marden 2006).

In most empirical studies, home range size is estimated using tracking methods such as direct observation, mark‐recapture, radio telemetry, and acoustic telemetry, depending on the species and habitat. These methods vary in spatial and temporal resolution but collectively provide a robust foundation for comparing space‐use behaviour across taxa.

To comprehensively disentangle the multifaceted variations in home range sizes and the factors influencing these relationships, a large‐scale comparative study is essential for identifying overarching patterns and assessing how ecological, behavioural, and physiological factors interact to shape home range size at different levels.

We aimed to assess the relationship between home range size and body size, as well as the relative influence of various biological and ecological factors including habitat type, social behaviour, migratory patterns, and trophic behaviour in home range variation across different aquatic taxa.

To this aim, and stemming from current knowledge on home range allometries and variations, we formulated and assessed a series of hypotheses. First, we expected that larger species in terms of body size would lead to higher levels of home range sizes, regardless of other species' traits (Jetz et al. 2004; Tamburello et al. 2015). Second, we predicted that body size interacted with energy‐demanding behavioural traits in shaping the size scaling of home range, across all taxa (e.g., Makarieva et al. 2005; Bejan and Marden 2006). Third, we postulated that key behavioural traits, independent of body size, can explain a portion of that variation which is not directly dependent on the allometric relationship with body size: in this case, we expected: (i) differences between migratory vs. non‐migratory species, with home range being larger for the former as per their distant and disjointed seasonal feeding areas during the annual cycle (e.g., thus animals must transit between these areas; Costa et al. 2016), compared to the latter which rely on more local trophic resources throughout the year; (ii) herbivore species, which feed on generally predictable, abundant, and widespread food resources (e.g., vegetation; Haskell et al. 2002), showing relatively smaller home ranges than carnivorous species (e.g., Kelt and van Vuren 2001; Broekman et al. 2024); (iii) pelagic species having larger home range than benthic ones, as animals that forage in three‐dimensional habitats usually face lower effective resource densities compared to animals that forage in two‐dimensional ones (e.g., Pawar et al. 2012; Tamburello et al. 2015); and (iv) sociability significantly affects space use, with individuals from group‐living species (shoaling) exploring larger areas to meet their minimum energy requirements compared to solitary species, due to the extensive inter‐individual overlap in available resources that characterizes shoaling species. As all these factors can synergistically influence variations in species' space use, disentangling their contribution will enhance our understanding of the evolutionary drivers of spatial behaviour and mechanisms of species interaction and coexistence.

To address this, we compiled a dataset from several published studies covering 172 species, spanning invertebrates, fish, reptiles, and mammals from freshwater to marine ecosystems, where home ranges were obtained using a range of animal tracking methods, including direct observation, mark‐recapture, and telemetry.

## 2 Materials and Methods

To address the aim of the study, we assembled a dataset on aquatic species' home range size from the published literature. We then recorded and classified the data according to key biological and ecological factors, including habitat type, social behaviour, migratory patterns, and trophic behaviour across different aquatic taxa.

### 2.1 Literature Search

A meta‐analysis of home range data of aquatic ecosystem species was conducted using two complementary approaches: (1) reviewing existing datasets of aquatic species' home range size (Minns 1995; Tamburello et al. 2015; Pinsky et al. 2019) and (2) conducting a systematic search of published and grey literature, including reports, project proceedings, and other similar sources.

For the literature search, we used Web of Science and Google Scholar through various keywords related to space‐use behaviour i.e., ‘animal movement’, ‘spatial ecology’, ‘individual home range’, ‘utilisation distribution’, ‘activity space’, ‘site fidelity’, ‘tracking’, ‘telemetry’, ‘mark’, ‘recapture’, ‘minimum convex polygon’ and ‘kernel density estimators’. Relevant sources cited within the publications were also examined. As a result of this investigation, we collected 190 papers published between 1971 and 2019. Most reported home range‐and body size‐averaged value, while a few provided individual‐level data. We included studies that provided at least a home range estimate for the specimens examined, and excluded studies that were purely descriptive, involved terrestrial species or lacked species‐level identification. After this selection, 97 papers were retained in the dataset utilised in the present work. When multiple studies reported data on the same species, we used the average of the available home range values.

### 2.2 Data Collection and Organisation

The data included in the present dataset are all at the species level. The species have been grouped into four macro taxonomic categories: invertebrates and vertebrates, the latter divided in turn into fish, reptiles, and mammals. Species were then subdivided into functional groups according to the classification available in controlled vocabularies (e.g., the LifeWatch ERIC thesauri https://www.lifewatch.eu/catalogue‐of‐services), species and species trait registers (e.g., FishBase http://www.fishbase.org/search.php; WoRMS http://marinespecies.org/; ADW https://animaldiversity.org/) and mentioned published papers. For each species, we compiled information on: (i) taxonomic group; (ii) vertical distribution (benthic and pelagic): species were classified as benthic if primarily associated with the substrate, including demersal species; pelagic species were those inhabiting the open water column. Subgroups such as demersal and coastal species were grouped accordingly, as our focus was on the dimensionality of space use; (iii) trophic level (carnivores and herbivores): omnivorous species were classified into one of these two categories based on their predominant dietary preference using FishBase or published literature; (iv) migratory behaviour (migratory and non‐migratory): species were classified as migratory if they exhibited long‐distance seasonal movement patterns, including anadromous and catadromous forms; (v) social behaviour (solitary and shoaling); (vi) individual home range area (m^2^); and (vii) individual body size, that was expressed as ash‐free dry weight ‘AFDW’ in g.

For example, the European eel ( *Anguilla anguilla* ) was classified as migratory and benthic; the Atlantic bluefin tuna ( *Thunnus thynnus* ) as migratory and pelagic; the brown surgeonfish ( *Acanthurus nigrofuscus* ) as herbivorous and benthic.

Moreover, for each study, we extracted the following: (i) biogeographical region; (ii) habitat type; (iii) tracking methods (direct observation, mark‐recapture, or telemetry). This latest background information was not expressed in the results, as we focused on a standardised tracking analysis.

As regards the individual body size, where not explicitly reported in the text, it was calculated from total individual length (mm) and published Length‐Weight Relationships (LWR) available on Fishbase. The weight was then transformed from wet weight to AFDW based on the mass conversion factor present in Fishbase (for fish) and scientific literature (for the other taxonomic groups).

Few studies included equal representation of both sexes; therefore, we were unable to conduct sex‐specific classification. We excluded several nematode groups from our analysis to minimise the occurrence of extreme body size outliers and unique benthic interstitial species in our dataset.

### 2.3 Home Range Estimation Methods

Home range estimates were derived from studies employing acoustic and radio telemetry, mark‐recapture techniques, and direct observation, depending on the species and ecosystem. These approaches differ in spatial and temporal resolution but collectively provide a robust basis for comparing space‐use behaviour across taxa.

The impact of the variation in the length of the tracking period was not considered, as this on the resulting home range and habitat selection estimates is rarely explicitly considered or taken into account (but see Börger et al. 2006). All original home range size data were expressed as *Minimum Convex Polygon* (MCP), *Kernel Density Estimator* (KDE) or both estimates. Considering that KDE was the most widely used home range estimator in the collected studies (70%), that seven studies reported both estimates, and that these estimates resulted strictly related (KDE = 0.29MCP + 14.51; *R* ^2^ = 0.90, *p* < 0.01), all home range estimates were converted to KDE values.

### 2.4 Data Analysis

Data for individual species were averaged to obtain single home range (m^2^) and body size (g) values for each species (e.g., McCauley et al. 2015). The sampled individuals were expected to be representative of the mean body size of individuals in these species, assuming size‐independent sampling (following Tamburello et al. 2015). To express body size‐home range area relationships as power laws (i.e., *Y* = *aX* ^b^), we log_10_‐transformed the variables home ranges (HR, m^2^) and body size (*M*, g AFDW). On the pooled data, the scaling of home range to body size was assessed using an ordinary least squares (OLS) regression model with log_10_ M as the explanatory variable. One‐way analysis of variance (one‐way ANOVA) was used to compare the average individual body size and average individual home range size among taxonomic levels. Linear analyses of covariance (ANCOVA) were used for pairwise analysis of the slopes and intercepts of home range scaling relationships across migratory and social behaviour, vertical distribution, and trophic level. More specifically, pairwise comparisons were conducted on the slopes of these relationships (when significant) among the taxonomic groups. In the case of non‐significant slopes, the same analysis was carried out on the intercepts, which represented the specific home range value for an individual of size equal to unity. Behavioural constraints were analysed on pooled data and within taxonomic levels.

### 2.5 Modelling Home Range Size Variation in Relation to Behavioural Traits

Because some behavioural traits alone, independent of body size and its allometric relationships with home range, could potentially explain a significant component of the total variability in home range size, we first extracted the residuals (i.e., the difference between the data observed values of the dependent variable and the fitted values) from a linear regression model between home range and body size. Then, we used them to model this variation at the species level, via generalised linear mixed models (GLMM; Zuur and Ieno 2016). In particular, we wanted to explore if the home range size variation beyond that already explained by body size (residuals) is related to the following key behavioural traits: migration (categorical: migratory vs. non‐migratory), food type (categorical: herbivore vs. carnivore), vertical distribution (categorical: benthic vs. pelagic) and sociability (categorical: shoaling vs. solitary), as well as their interactions. A preliminary data exploration suggested that moderate multicollinearity (VIF > 5) was found for the interaction between migration and sociability. If two or more predictors are correlated with each other, the fit of the model will be compromised; thus, the removal of the interactions between predictors with the largest VIF is required. Accordingly, the interactive effects between the aforementioned predictor variables were not included but treated as additive effects in the model. The interactive effect was kept for all other combinations of predictors. The residuals of the relationship between home range and body size were set as the response variable and modelled with Tweedie errors (link = log), as it is customary with continuous, 0+ variables (Zuur et al. 2009). In all models, we included ‘species’ as a random effect. For each model set, we fitted candidate models comprising all potential combinations of predictors, also including the null model, because each combination could represent a distinct a priori hypothesis. The model selection used Akaike's Information Criterion corrected for small sample sizes (AICc). Models were selected if they had ΔAICc ≤ 2, and if their AICc value was lower than that of any simpler alternative (Harrison et al. 2018). Therefore, we obtained either a set of top‐ranked models or a single best model for the response variable. Inference about the effects of predictors was made using the best model: we estimated coefficients and 95% confidence intervals (CIs) of predictors, assessing whether 95% CIs overlapped ‘0’ to identify informative predictors (see Table S1 for the full model selection output). The result of model selections GLMMs and model selections were performed in R 4.3.1, using the function *glmmTMB::glmmTMB* (Brooks et al. 2017) for fitting models and *dredge::MuMIn* (Bartoń 2020) for model selection. Best models were validated through visual inspection of residuals through the ‘DHARMa’ package (Hartig and Hartig 2022).

## 3 Results

### 3.1 Dataset Composition and Taxonomic Overview

The final dataset included 172 aquatic species, comprising 4 invertebrates, 136 fish, 10 reptiles, and 22 mammals. Invertebrates ranged in body size from −1.51 to 1.83 log_10_ g AFDW and in home range size from −1.46 to 4.27 log_10_ m^2^. Fish species ranged from −1.30 to 4.06 log_10_ g AFDW and −1.52 to 10.26 log_10_ m^2^. Reptiles ranged between 2.33 and 4.69 log_10_ g AFDW and from 5.38 to 9.88 log_10_ m^2^. Mammals ranged from 3.74 to 7.20 log_10_ g AFDW and 6.56 to 11.06 log_10_ m^2^.

**FIGURE 1:**
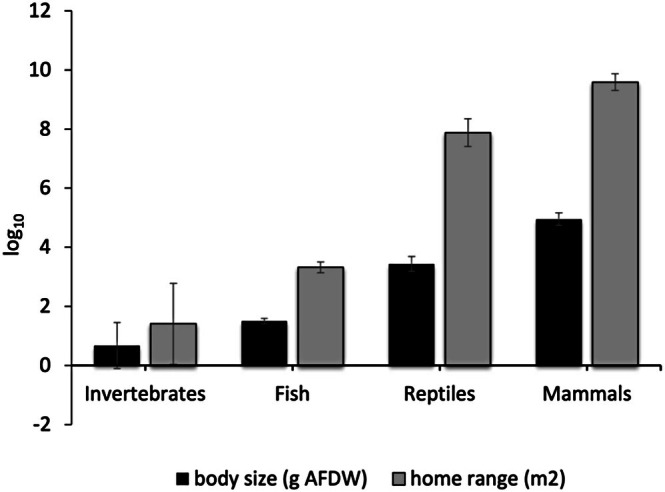
Variation in average individual body sizes and home range across main taxonomic groups. Body sizes range from 0.67 ± 0.78 g to 4.95 ± 0.21 g, while home ranges vary from 1.39 ± 1.47 m^2^ to 9.59 ± 0.28 m^2^.

Average individual body sizes significantly varied across main taxonomic groups: 0.67 ± 0.78 (g, AFDW) for invertebrates, 1.50 ± 0.09 (g, AFDW) for fish, 3.44 ± 0.25 (g, AFDW) for reptiles, and 4.95 ± 0.21 (g, AFDW) for marine mammals (one‐way ANOVA: *F*_3,168_ = 74.43, *p* < 0.001; Figure 1). Similarly, average individual home ranges also significantly varied across the main taxonomic groups: 1.39 ± 1.47 (m^2^) for invertebrates, 3.32 ± 0.18 (m^2^) for fish, 8.08 ± 0.47 (m^2^) for reptiles, and 9.59 ± 0.28 (m^2^) for marine mammals (one‐way ANOVA: *F*_3,168_ = 73.32, *p* < 0.001; Figure 1).

### 3.2 Allometric Scaling of Home Range

**FIGURE 2:**
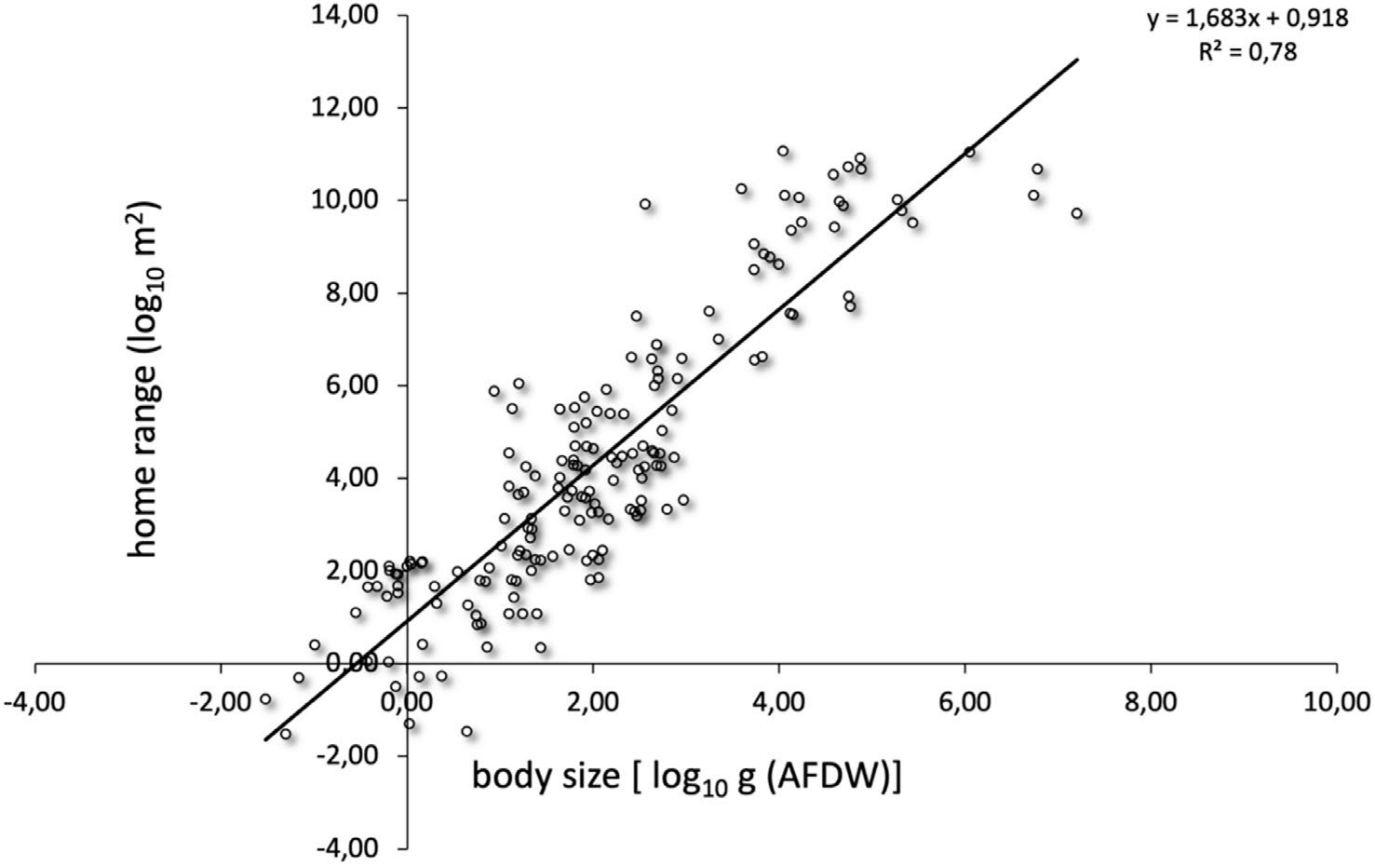
The allometric scaling relationship between average individual body size and average individual home range.

**FIGURE 3:**
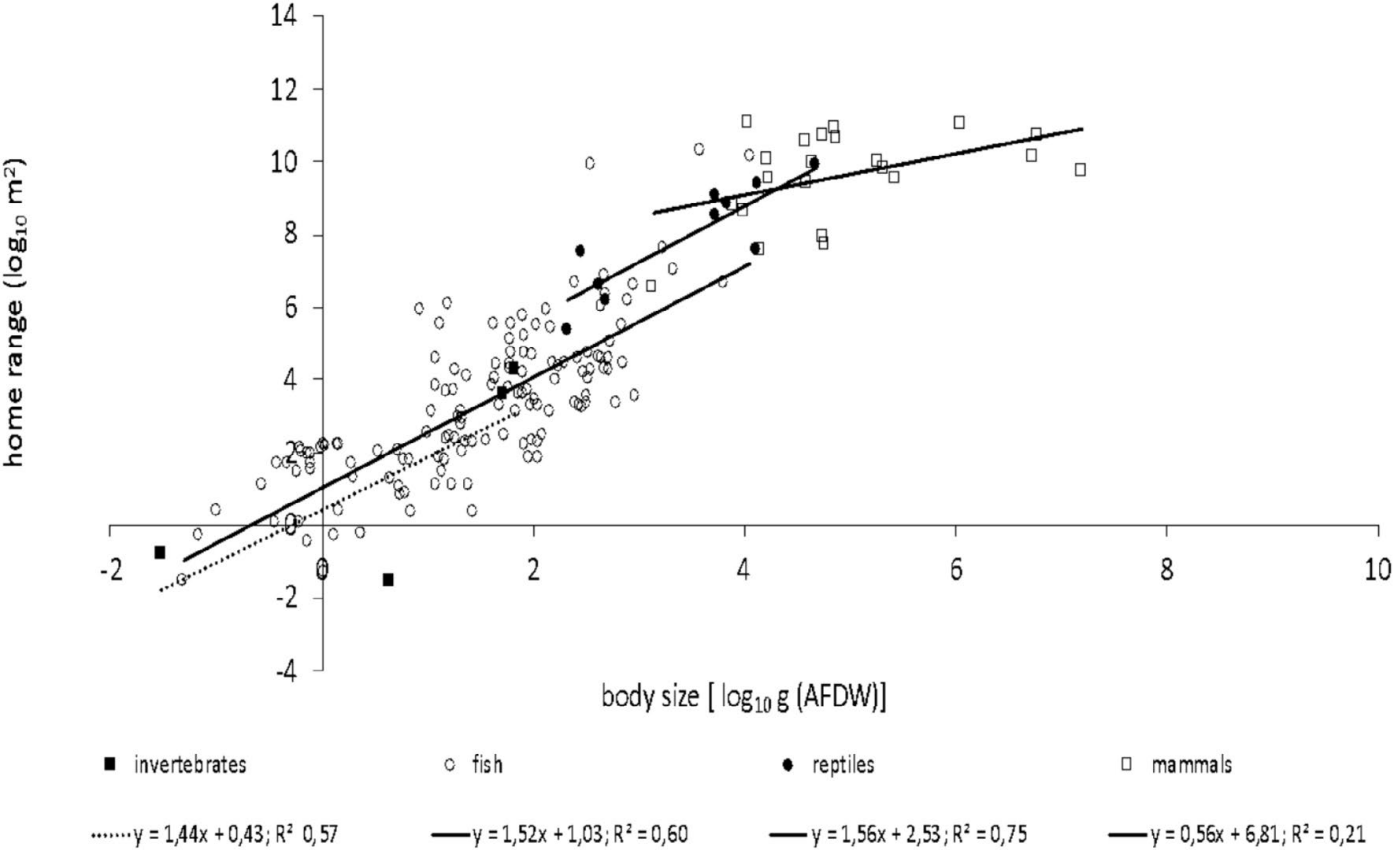
The scaling relationship between average individual home range and average individual body size across taxonomic groups.

Overall, average individual home range scaled with average individual body size, with a scaling exponent of 1.68 ± 0.07 and a scaling coefficient/intercept of 0.92 (Figure 2), and body size explained 78% of the variation in home range size. At the inter‐taxonomic level, the relationship between home range size and body size was significant in all taxonomic groups except for invertebrates. Home range size scaled with an exponent of 1.52 ± 0.11 (*F*_1,134_ = 197.9; *p* < 0.001) for fish; 1.56 ± 0.32 (*F*_1,8_ = 24.28; *p* = 0.001) for reptiles; and 0.56 ± 0.26 (*F*_1,20_ = 3.94; *p* = 0.041) for mammals (Figure 3). Since the relationship of home range size against body size for invertebrates was not significant, they were excluded from the pairwise comparison with the other taxonomic groups. The scaling exponent of the relationship between home range size and body size in mammals was significantly lower than in other taxonomic groups (Table 1). The scaling exponents of the relationship between home range size and body size did not differ between fish and reptiles, while the scaling coefficients (intercepts) in reptiles were significantly higher than in fish (*F*_1,143_ = 11.15; *p* = 0.001) (Table 1).

**TABLE 1:** Regression parameters and pairwise comparison among taxonomic groups regarding the relationship between home range size and body size. The allometric relationship between body size and home range was not significant for invertebrates, thus they were excluded from the pairwise comparison. The *n* represents the sample number, *b* and *a* denote the slope and the intercept respectively and *R* ^2^ is the coefficient of determination.

Regression parameters	*n*	*b*	*a*	*p*	*R* ^2^	Intergroup comparison	*b*1 = *b*2	*a*1 = *a*2
							0.022	
Invertebrates	4	1.44 ± 0.88	0.44 ± 1.30	0.241	0.58	Fish vs. mammals	0.002	
Fish	136	1.52 ± 0.11	1.03 ± 0.20	< 0.001	0.60	Fish vs. reptiles	0.951	0.001
Reptiles	10	1.56 ± 0.32	2.53 ± 1.12	0.001	0.75	Mammals vs. reptiles	0.045	
Mammals	22	0.56 ± 0.26	6.82 ± 1.33	0.041	0.21	Mammals vs. reptiles	0.045	

Species energy requiring functional traits explained a significant component (27.15%) of the variation unexplained by species average body size, with specific contributions as follows: 17.61% by vertical distribution; 6.59% by trophic level; 2.95% by migration.

### 3.3 Behavioural Traits

#### 3.3.1 Vertical Habitat Use

**FIGURE 4:**
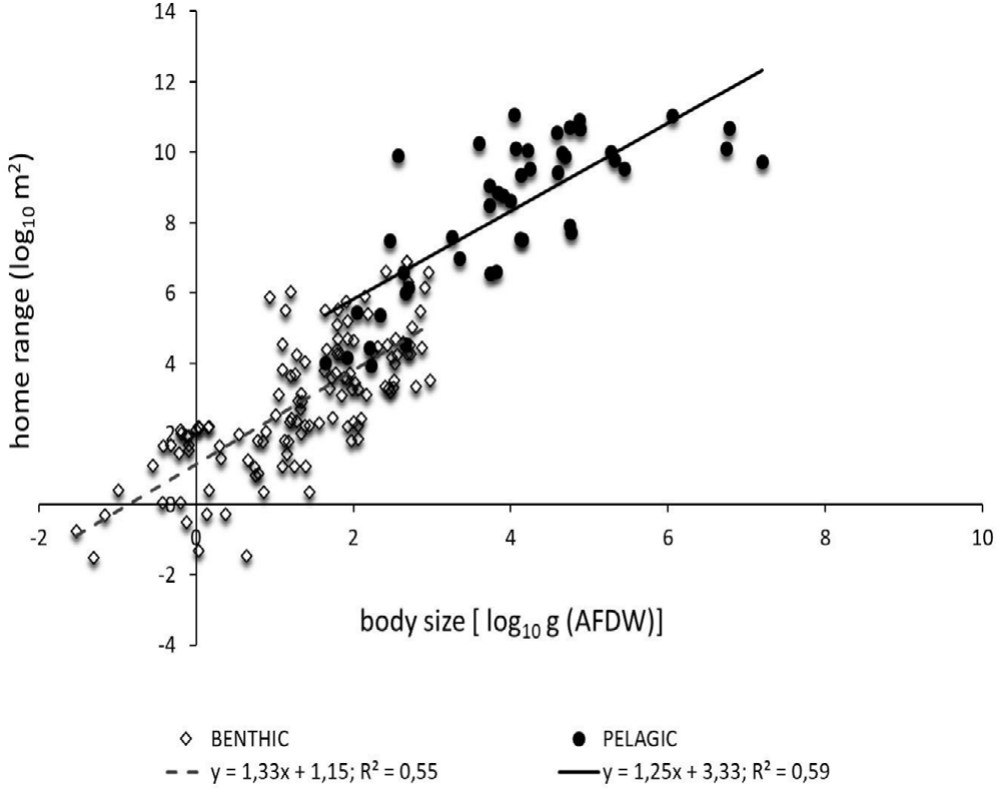
The allometric scaling relationships between home range and body size across different vertical distribution behaviours.

Species were grouped according to their position in the water column, reflecting two distinct vertical habitat types: benthic (bottom‐dwelling) and pelagic (open‐water). The dataset included 127 benthic species and 45 pelagic species. The benthic group comprised 123 fish and 4 invertebrates, with no reptiles or mammals present. The pelagic species included 13 fish, 10 reptiles, and 22 mammals, with no invertebrates present. The relationship between home range and body size was highly significant for benthic (*F*_1,125_ = 151.2, *p* < 0.001) and pelagic species (*F*_1,43_ = 61.14, *p* < 0.001). Species' home range scaled with body size with a scaling exponent of 1.33 for the benthic species and 1.25 for the pelagic ones (*F*_1,168_ = 0.19, *p* = 0.665), while the benthic species showed a significantly lower intercept than the pelagic ones (benthic 1.51 ± 0.18; pelagic 3.33 ± 0.67; *F*_1,169_ = 35.51, *p* < 0.0001) (Figure 4).

#### 3.3.2 Trophic Category

**FIGURE 5:**
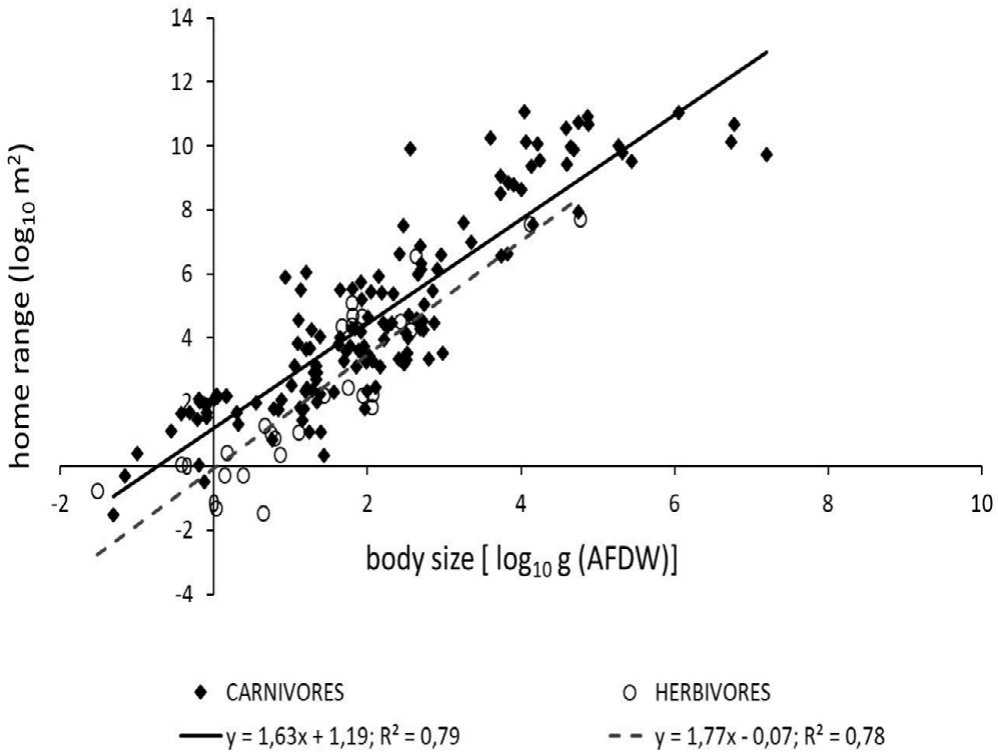
The allometric scaling relationships between body size and home range across different trophic behaviours.

In the pooled data, 145 carnivore species and 27 herbivore ones were included. Carnivorous species consisted of 2 invertebrate species, 114 fish, 8 reptiles, and 21 mammals. Among the herbivores, there were 2 invertebrate species, 22 fish, 2 reptiles, and one mammal species, the *dugong Dugong dugon*. Allometric relationships between home range and body size were significantly different for both carnivorous (*F*_1,143_ = 528.1, *p* < 0.001) and herbivorous species (*F*_1,25_ = 90.52, *p* < 0.001). Home range size scaled with body size with an exponent of 1.63 for carnivores and 1.77 for herbivores. The scaling exponents were not significantly different between these two groups (*F*_1,168_ = 0.434, *p* = 0.511). Carnivores showed a significantly higher intercept (*a* = 1.19 ± 0.19) compared to herbivores (*a* = −0.07 ± 0.36) (*F*_1,169_ = 13.06, *p* < 0.001) (Figure 5).

#### 3.3.3 Migratory Behaviour

**FIGURE 6:**
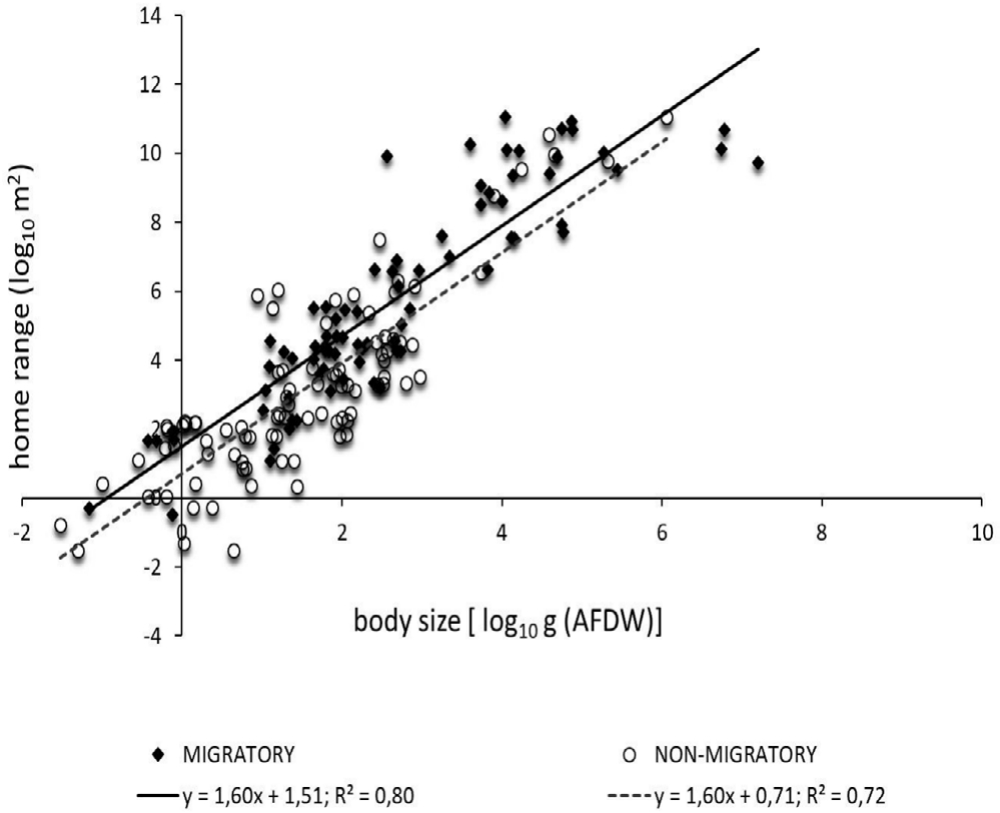
The allometric scaling relationships between body size and home range for migratory and non‐migratory behaviour.

On the pooled data, there were 81 migratory species and 91 non‐migratory ones. The migratory species were constituted by 2 invertebrate species ( *Palinurus elephas* and *Homarus gammarus* ), 56 fish, 8 reptiles, and 15 mammals. Non‐migratory species were divided as follows: 2 invertebrates, 80 fish, 2 reptiles, and 7 mammals. Allometric relationships between body size and home range were highly significant for migratory (*F*_1,79_ = 315.6, *p* < 0.001) and non‐migratory species (*F*_1,89_ = 225.0, *p* < 0.001). The scaling exponent of migratory and non‐migratory species was not significantly different (*F*_1,164_ = 0.167, *p* = 0.475), while the intercept corresponding to specific home range surface area was lower for migratory than non‐migratory species (ANCOVA, *F*_1,169_ = 12.86, *p* = 0.004) (Figure 6).

#### 3.3.4 Social Behaviour

**FIGURE 7:**
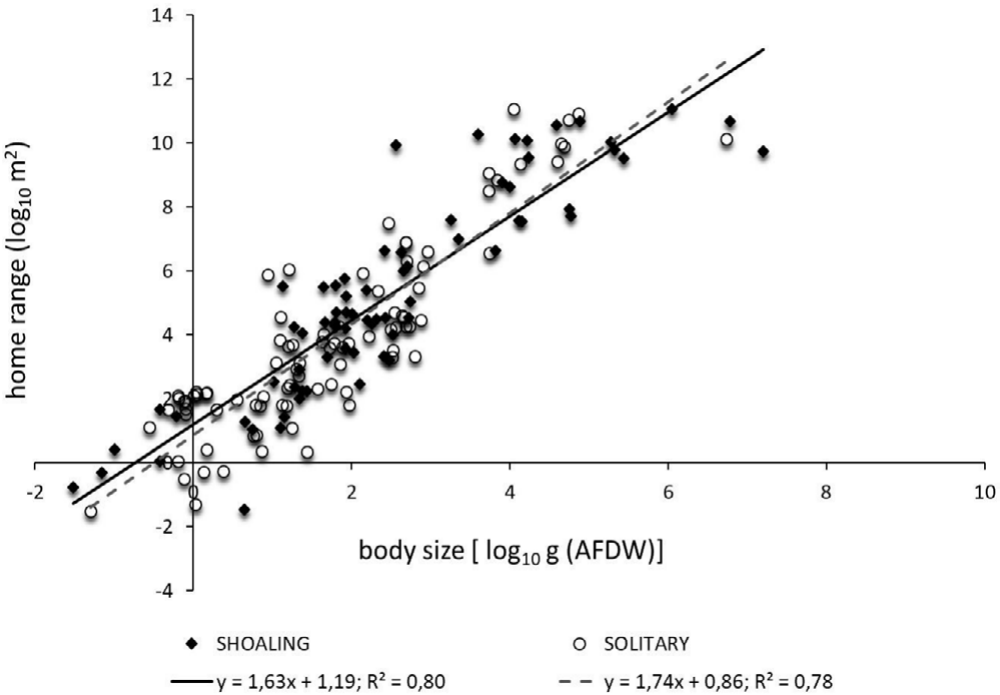
The allometric scaling relationships between body size and home range in relation to shoaling or solitary behaviour.

The species were divided into‘shoaling’ r‘solitary’ In the dataset, there were 72 shoaling species divided as follows: 2 invertebrates, 52 fish, 3 reptiles, and 15 mammals. Solitary species were 89: 2 invertebrates, 73 fish, 7 reptiles, and 7 mammals. Data for 11 fish species, which could not have been allocated to either shoaling or solitary categories, were not considered in the following analysis. The allometric relationship between average individual body size and average individual home range was significant for both social behaviour types (Figure 7). Home range size scaled with body size with an exponent of 1.63 (*F*_1,70_ = 285.9, *p* < 0.001) for shoaling species and with an exponent of 1.74 (*F*_1,87_ = 315.2, *p* < 0.001) for solitary species. Neither the scaling exponent of the relationship between home range and body size in shoaling and solitary groups (*F*_1,157_ = 0.627, *p* = 0.429), nor the scaling coefficient/intercept, were significantly different (*F*_1,158_ = 0.243, *p* = 0.622) (Figure 7).

#### 3.3.5 Integrated Contribution of Behavioural Traits to Home Range Variations

Beyond body size, we found that specific behavioural traits contributed to home range size variation in aquatic organisms. Considering the relative importance of the variables, three best models (supporting similar effects) were identified as the most parsimonious based on ΔAICc (values less than 2) for home range size (Table S1). The best‐supported model revealed that, across all taxonomic groups, home range size was significantly larger for carnivorous and pelagic species (Table 2; Table S1). Model selection did not retain any significant interactive effect between these predictors. In synthesis, species with pelagic behaviour and/or carnivore food type showed on average significantly larger home range size than benthic and/or herbivore species. Despite its significant role when interacting with body size (see previous results), migration was not retained by model selection as a standalone predictor significantly influencing home range size. Similarly, the social behaviour predictor, i.e., solitary vs. shoaling species, was not retained by the model selection, as this trait showed no significant association with variation in home range size (Table 2; see Table S1 for a full list of results on compared models).

**TABLE 2:** Parameters estimated from the top‐ranked Generalised Linear Mixed Models (GLMMs) predicting the size‐adjusted home range (i.e., the residual variation in home range after excluding the effects of body size), in relation to behavioural traits, include: Variance of random intercepts (*σ* ^2^), predictors' coefficient estimates (*β*), and their 95% confidence intervals (CIs). Coefficients whose CIs do not include ‘0’ are reported in bold.

Model anking	Response variable	Predictor	*β* coefficient	95% CI
1	Residuals of the hr ~ bs relationship (log_10_) *σ* ^2^ Individual = 1.36 (best model)	Intercept	−0.7054	−1.0018601; −0.4089904
		Foodtype: herbivore	−1.2076	**−2.1242996**; **−0.2908940**
		Vertical distribution: pelagic	0.6882	**0.2304572**; **1.1459146**
2	Residuals of the hr ~ bs relationship (log_10_) *σ* ^2^ Individual = 1.35 (second best model)	Intercept	−0.6028	−1.0011681; −0.2044637
		Migration: non‐migratory	−0.1549	−0.6436062; 0.3338098
		Foodtype: herbivore	−0.6430	−1.7705305; 0.4845714
		Vertical distribution: pelagic	0.5992	**0.1179353**; **1.0804960**
		Foodtype: herbivore × Migration: non‐migratory	−1.5282	−3.5547830; 0.4983699
3	Residuals of the hr ~ bs relationship (log_10_) *σ* ^2^ Individual = 1.36 (third best model)	Intercept	−0.5691	−0.9656479; −0.1726084
		Foodtype: herbivore	−1.2155	**−2.1374526**; **−0.2936201**
		Migration: non‐migratory	−0.2417	−0.7203707; 0.2369414
		Vertical distribution: pelagic	0.6057	**0.1211241**; **1.0901832**

## 4 Discussion

Individuals interact within shared space where the home ranges of two or more individuals, either of the same or different species, overlap. Therefore, extending the knowledge on factors affecting the spatial dimension of individual home ranges is essential to deepen the understanding of changing interactive networks and the biodiversity organisation.

### 4.1 Body Size Scaling of Home Range

Individual home range size is known to scale with individual body size (McNab 1963), with a scaling exponent greater than expected from the allometric scaling of individual metabolic rate, which typically scales with body mass to the power of 2/3 to 3/4 (Lindstedt et al. 1986; Harestad and Bunnel 1979; Haskell et al. 2002; Jetz et al. 2004; Glazier 2005). The positive relationship between body mass and home range size reflects that larger species generally have higher absolute energetic demands and thus require access to more spatially distributed resources, necessitating broader home ranges to meet their energy requirements. Fractal resource distribution (Haskell et al. 2002) and intra‐specific overlap in individual home ranges (Jetz et al. 2004) have been proposed to affect the scaling coefficient of the individual home range size. The former hypothesis would imply an inverse body size scaling of perceived resource availability, resulting in a decreasing resource exploitation efficiency with increasing individual body size (Haskell et al. 2002; Basset et al. 2012; Cozzoli et al. 2020).

Our results are consistent with these classical frameworks, reinforcing the role of body size as a key determinant of individual home range size (McNab 1963), and aligning with subsequent seminal studies (Lindstedt et al. 1986; Harestad and Bunnel 1979; Minns 1995). It has been suggested that the size scaling exponent is similar to that of the universal allometric scaling of metabolic rates, which remains actively debated (e.g., Tamburello et al. 2015; Udyawer et al. 2022). In this study, the size scaling of the home range has been shown to vary from hypo‐allometric to hyper‐allometric. This finding aligns with the current study of Udyawer et al. (2022), who have demonstrated that size scaling varies across taxa, and with Minns (1995) who showed that the home range scaling exponent can vary from hypo‐allometric to hyper‐allometric depending on the habitat. Furthermore, the recent studies underscored that, while body mass—a chief determinant of metabolic rate—significantly influences space use, variation in metabolic rate phenotypes independent of body size also contributes substantially to differences in space use (Shokri et al. 2024, 2025).

### 4.2 Influence of Behavioural Traits on Home Range

Our results support seminal studies in size scaling of home range, while it extends beyond taxonomic boundaries to highlight the significant influence of energy‐expensive behavioural traits on home range dynamics. This underscores the relative importance of both individual traits and behavioural traits in understanding spatial ecology. We observed that home range variation, beyond body size, is mainly driven by mechanisms influencing the effective distribution of trophic resources, specifically, vertical habitat uses and trophic level. Overall, behavioural traits, beyond body size, accounted for 27.15% of the variation in home range size. A species' diet type (Jetz et al. 2004) and the dimensionality of its foraging environment have both been suggested as important determinants of space‐use scaling (e.g., benthic vs. pelagic; Carbone et al. 2007; Heit et al. 2023).

Moreover, migratory behaviour was associated with larger home ranges compared to non‐migratory species. However, this influence was only marginal once body size‐independent variation was accounted for, and migratory behaviour did not show a statistically significant effect. Several factors could explain this result. First, behavioural studies on aquatic migratory species are often limited by technological constraints that restrict temporal and spatial resolution, possibly leading to underestimates of their home range sizes. Second, trophic level has been shown to influence individual home range size, with carnivore species showing a larger individual home range size than herbivore ones also when body size‐independent variations were considered. At higher trophic levels, the losses of energy associated with the capture, digestion, and utilisation of resources often reduce the availability of food resources per unit area. This leads to larger home range sizes for carnivores, as they need to cover more area to meet their energy requirements due to much lower resource densities compared to herbivores (Haskell et al. 2002). This result is well supported by several empirical studies (e.g., Lindstedt et al. 1986; Hirt et al. 2021; Tamburello et al. 2015). Third, we found that vertical habitat use plays an important role, with pelagic species requiring larger home ranges than benthic ones, independent of body size. This indicates that foraging in three‐dimensional environments influences individual space use more than two‐dimensional foraging does (Haskell et al. 2002). Pelagic species, operating in a larger search space, face lower resource densities and therefore require wider foraging areas (e.g., Pawar et al. 2012; Tamburello et al. 2015). These results contribute to our understanding of how horizontal and vertical behavioural patterns affect resource partitioning and species coexistence. Fourth, regardless of body size, solitary and shoaling behaviour showed no systematic influence on home range size. Shoaling organisms, while not closely following each other, tend to move to similar general areas, utilising different parts of their home ranges. Sociality in group‐living species, characterised by encounters and shared space use, influences movement patterns and resource competition dynamics (Pillans et al. 2017; Allen and Singh 2016; Monti et al. 2024). Increased encounter rates with larger group sizes suggest intensified intra‐population resource competition, although complex habitats may disrupt group cohesion, affecting shared space utilisation (Cross et al. 2013; Haydon et al. 2008; Vander Wal et al. 2014).

### 4.3 Methodological Robustness and Limitations

In this context, and in line with the central objectives of this study, our results on aquatic species show that: (1) energy‐expensive behavioural traits directly influence individual home‐range size, independently of taxonomic constraints or specificity and (2) behavioural traits related to intra‐specific spatial overlap, used as a proxy for niche overlap, do not appear to affect the allometric scaling of home‐range size. The results obtained do not appear to be influenced by methodological biases, either in the quantification of individual home‐range size or in the assignment of taxa to behavioural functional groups. Home‐range estimates are often derived using diverse methodologies and techniques, each based on specific assumptions and modeling approaches that may introduce the risk of under‐ or over‐estimation (Kie et al. 2010; Silva et al. 2021; Udyawer et al. 2022).

While meta‐analyses using data from repositories, published datasets, or literature searches are prone to such biases, they typically implement strategies to minimize inaccuracies. However, the findings of this study do not appear to be affected by the specific methodologies used to estimate species' home ranges in the original sources. This robustness is supported, first, by the comparative design underpinning the hypothesis testing, which focused on differences in average home range size across functional traits (e.g., migratory vs. non‐migratory, carnivorous vs. herbivorous, pelagic vs. benthic, and shoaling vs. solitary species), rather than on absolute home range values, thereby reducing methodological inconsistency. Second, the statistical analysis of body size‐independent variation in home range size attributed to functional traits helped remove potential biases due to interactions between body size and space‐use behaviour. Third, the close similarity in scaling factors observed across taxonomic groups, except for mammals, indicates that the patterns in home range size variation across behavioural traits are unlikely to be driven by uneven taxonomic representation in the dataset. Many space‐use models assume the existence of home ranges a priori, rather than treating them as emergent properties of animal movement. Moreover, mechanistic movement models often fail to produce home range behaviour unless constrained by *ad hoc* mechanisms, such as reflective boundaries or site fidelity (Börger et al. 2008). In contrast, our findings demonstrate that both body size and individual behavioural traits influence home range size. For example, larger species are able to travel farther than smaller ones due to their higher absolute energetic demands, which require access to broader spatial areas to meet resource needs (McNab 1963; Shokri et al. 2025). Body size was the primary driver of variation in home range size across aquatic species, while energy‐demanding behavioural traits further explained a substantial portion of the remaining variation, with notable differences among trait categories.

## 5 Conclusion

The comparative analysis conducted in this study on the size‐independent impact of behavioural traits on space use allows extending the study result to an analysis of their implication at the ecosystem level. To this end, our study contributes to advance our understanding of the drivers of spatial use in aquatic species by demonstrating that, while body size has well‐established effects, functional behavioural traits beyond body size, such as trophic level and vertical habitat use, significantly contribute to additional variation in home range size across species. By shaping how individuals use space, these traits may directly affect the density and, potentially, the intensity of intra‐ and inter‐specific interactions at the community and ecosystem levels with important implications for the competitive coexistence mechanisms (Haskell et al. 2002; Basset et al. 2012; Basset and Angelis 2007). Such behaviours often entail energetic demands and may ultimately be reflected in metabolic rate, potentially offering, as suggested by (Shokri et al. 2024), a more holistic descriptor of foraging and space use behaviour across taxa. Given the ecological and evolutionary relevance of spatial behaviour, especially in relation to species interactions, resource partitioning, and biodiversity organisation, future research should aim to incorporate functional behavioural traits and metabolic rate into spatial ecological models alongside body size.

## Author Contributions

**Vanessa Marrocco:** conceptualization (lead), data curation (lead), formal analysis (lead), investigation (lead), methodology (lead), writing – original draft (lead), writing – review and editing (lead). **Milad Shokri:** formal analysis (supporting), investigation (supporting), writing – review and editing (equal). **Flavio Monti:** data curation (supporting), formal analysis (equal), writing – review and editing (equal). **Francesco Cozzoli:** writing – review and editing (supporting). **Fabio Vignes:** writing – review and editing (supporting). **Alberto Basset:** conceptualization (lead), funding acquisition (lead), supervision (lead), writing – review and editing (equal).

## Conflicts of Interest

The authors declare no conflicts of interest.

## Supporting information


Table S1.

## Data Availability

The data are accessible for peer review via the Open Science Framework at https://osf.io/sc5tr/?view_only=c523bd2245e5444dbbe0b98381c8f14. The data is also permanently stored in LifeWatch Italy data repository.

## References

[ece371886-bib-0073] Allen, A. M. , and N. J. Singh . 2016. “Linking Movement Ecology With Wildlife Management and Conservation.” Frontiers in Ecology and Evolution 3: 155. 10.3389/fevo.2015.00155.

[ece371886-bib-0072] Bartoń, K. 2020. “MuMIn: Multi‐Model Inference (Version 1.43.17).” CRAN. https://CRAN.R‐project.org/package=MuMIn.

[ece371886-bib-0001] Basset, A. 1995. “Body Size‐Related Coexistence: An Approach Through Allometric Constraints on Home‐Range Use.” Ecology 76: 1027–1035.

[ece371886-bib-0002] Basset, A. , and D. L. Angelis . 2007. “Body Size Mediated Coexistence of Consumers Competing for Resources in Space.” Oikos 116: 1363–1377.

[ece371886-bib-0003] Basset, A. , F. Cozzoli , and F. Paparella . 2012. “A Unifying Approach to Allometric Scaling of Resource Ingestion Rates Under Limiting Conditions.” Ecosphere 3: 1–14.

[ece371886-bib-0004] Bejan, A. , and J. H. Marden . 2006. “Constructing Animal Locomotion From New Thermadynamics Theory: Although Running, Flying and Swimming Appear to Be Distinctly Different Types of Movement, They May Have Underlying Physics in Common.” American Scientist 94: 342–349.

[ece371886-bib-0007] Börger, L. , B. D. Dalziel , and J. M. Fryxell . 2008. “Are There General Mechanisms of Animal Home Range Behaviour? A Review and Prospects for Future Research.” Ecology Letters 11: 637–650. 18400017 10.1111/j.1461-0248.2008.01182.x

[ece371886-bib-0008] Börger, L. , N. Franconi , F. Ferretti , et al. 2006. “An Integrated Approach to Identify Spatiotemporal and Individual‐Level Determinants of Animal Home Range Size.” American Naturalist 168: 471–485. 10.1086/507883 17004219

[ece371886-bib-0009] Broekman, M. J. , J. P. Hilbers , S. Hoeks , M. A. Huijbregts , A. M. Schipper , and M. A. Tucker . 2024. “Environmental Drivers of Global Variation in Home Range Size of Terrestrial and Marine Mammals.” Journal of Animal Ecology 93, no. 4: 488–500. 38459628 10.1111/1365-2656.14073

[ece371886-bib-0010] Brooks, M. E. , K. Kristensen , K. J. Van Benthem , et al. 2017. “glmmTMB Balances Speed and Flexibility Among Packages for Zero‐Inflated Generalized Linear Mixed Modeling.” R Journal 9: 378–400.

[ece371886-bib-0011] Brown, J. H. , J. F. Gillooly , A. P. Allen , V. M. Savage , and G. B. West . 2004. “Toward a Metabolic Theory of Ecology.” Ecology 85: 1771–1789.

[ece371886-bib-0069] Brown, J. S. , and B. P. Kotler . 2004. “Hazardous Duty Pay and the Foraging Cost of Predation.” Ecology Letters 7, no. 10: 999–1014. 10.1111/j.1461-0248.2004.00661.x.

[ece371886-bib-0012] Burt, W. H. 1943. “Territoriality and Home Range Concepts as Applied to Mammals. Source.” Journal of Mammalogy 24: 346.

[ece371886-bib-0013] Byrnes, E. E. , J. L. Hounslow , V. Heim , et al. 2023. “Intraspecific Scaling of Home Range Size and Its Bioenergetic Dependence.” *bioRxiv*. 2023‐06. 10.1002/ecy.70003 PMC11800060 39912258

[ece371886-bib-0015] Carbone, C. , A. Teacher , and J. M. Rowcliffe . 2007. “The Costs of Carnivory.” PLoS Biology 5: e22. 17227145 10.1371/journal.pbio.0050022 PMC1769424

[ece371886-bib-0016] Costa, B. M. , F. Polgar , and K. E. Limburg . 2016. “The Spatial Ecology of Anadromous Fishes: Migration, Habitat Use, and Implications for Conservation.” Canadian Journal of Fisheries and Aquatic Sciences 73: 123–135.

[ece371886-bib-0017] Cozzoli, F. , M. Shokri , G. Ligetta , F. Vignes , and A. Basset . 2020. “From Resource Exploitation to Animal Movement: A General Framework for the Spatial Analysis of Foraging Behaviour.” Oikos 129: 691–707. 10.1111/oik.07378.

[ece371886-bib-0018] Cross, P. C. , T. G. Creech , M. R. Ebinger , et al. 2013. “Female Elk Contacts Are Neither Frequency nor Density Dependent.” Ecology 94: 2076–2086. 24279278 10.1890/12-2086.1

[ece371886-bib-0020] Edelsparre, A. H. , M. J. Fitzpatrick , M. A. Rodríguez , and M. B. Sokolowski . 2021. “Tracking Dispersal Across a Patchy Landscape Reveals a Dynamic Interaction Between Genotype and Habitat Structure.” Oikos 130, no. 1: 79–94.

[ece371886-bib-0024] Glazier, D. S. 2005. “Beyond the ‘3/4‐Power Law’: Variation in the Intra‐ and Interspecific Scaling of Metabolic Rate in Animals.” Biological Reviews 80: 611–662. 10.1017/S1464793105006834. 16221332

[ece371886-bib-0027] Harestad, A. S. , and F. L. Bunnel . 1979. “Home Range and Body Weight—A Reevaluation.” Ecology 60: 389–402.

[ece371886-bib-0028] Harrison, X. A. , L. Donaldson , M. E. Correa‐Cano , et al. 2018. “A Brief Introduction to Mixed Effects Modelling and Multi‐Model Inference in Ecology.” PeerJ 6: e4794. 29844961 10.7717/peerj.4794 PMC5970551

[ece371886-bib-0029] Hartig, F. , and M. F. Hartig . 2022. “Package ‘DHARMa.’ R Package.” Accessed 5 September 2022. https://CRAN.R‐project.org/package=DHARMa.

[ece371886-bib-0030] Haskell, J. P. , M. E. Ritchie , and H. Olff . 2002. “Fractal Geometry Predicts Varying Body Size Scaling Relationships for Mammal and Bird Home Ranges.” Nature 418: 527–530. 12152078 10.1038/nature00840

[ece371886-bib-0031] Haydon, D. T. , J. M. Morales , A. Yott , D. A. Jenkins , R. Rosatte , and J. M. Fryxell . 2008. “Socially Informed Random Walks: Incorporating Group Dynamics Into Models of Population Spread and Growth.” Proceedings of the Royal Society B: Biological Sciences 275: 1101–1109. 10.1098/rspb.2007.1688 PMC2600907 18270158

[ece371886-bib-0032] Heit, D. R. , C. C. Wilmers , W. Ortiz‐Calo , and R. A. Montgomery . 2023. “Incorporating Vertical Dimensionality Improves Biological Interpretation of Hidden Markov Model Outputs.” Oikos 2023: e09820.

[ece371886-bib-0033] Hendriks, A. J. 2007. “The Power of Size: A Meta‐Analysis Reveals Consistency of Allometric Regressions.” Ecological Modelling 205: 196–208.

[ece371886-bib-0034] Hirt, M. R. , A. D. Barnes , A. Gentile , et al. 2021. “Environmental and Anthropogenic Constraints on Animal Space Use Drive Extinction Risk Worldwide.” Ecology Letters 24: 2576–2585. 34476879 10.1111/ele.13872

[ece371886-bib-0036] Jetz, W. , C. Carbone , J. Fulford , and J. H. Brown . 2004. “The Scaling of Animal Space Use.” Science 306: 266–268. 15472074 10.1126/science.1102138

[ece371886-bib-0037] Kelt, D. A. , and D. H. van Vuren . 2001. “The Ecology and Macroecology of Mammalian Home Range Area.” American Naturalist 157: 637–645. 10.1086/320621 18707280

[ece371886-bib-0074] Kie, J. G. , J. Matthiopoulos , J. Fieberg , et al. 2010. “The Home‐Range Concept: Are Traditional Estimators Still Relevant With Modern Telemetry Technology?” Philosophical Transactions of the Royal Society, B: Biological Sciences 365, no. 1550: 2221–2231. 10.1098/rstb.2010.0093. PMC2894967 20566499

[ece371886-bib-0038] Kotler, B. P. , J. S. Brown , and A. Subach . 1993. “Mechanisms of Species Coexistence of Optimal Foragers: Temporal Partitioning by Two Species of Sand Dune Gerbils.” Oikos 67, no. 3: 548–556. 10.2307/3545367.

[ece371886-bib-0071] Lindstedt, S. L. , B. J. Miller , and S. W. Buskirk . 1986. “Home Range, Time, and Body Size in Mammals.” Ecology 67, no. 2: 413–418. 10.2307/1938584.

[ece371886-bib-0068] MacArthur, R. H. , and E. R. Pianka . 1966. “On Optimal Use of a Patchy Environment.” American Naturalist 100, no. 916: 603–609. 10.1086/282454.

[ece371886-bib-0070] Makarieva, A. M. , V. G. Gorshkov , and B. L. Li . 2004. “Body Size, Energy Consumption and Allometric Scaling: A New Dimension in the Diversity–Stability Debate.” Ecological Complexity 1, no. 2: 139–175. 10.1016/j.ecocom.2004.02.003.

[ece371886-bib-0039] Makarieva, A. M. , V. G. Gorshkov , and B.‐L. Li . 2005. “Why Do Population Density and Inverse Home Range Scale Differently With Body Size?: Implications for Ecosystem Stability.” Ecological Complexity 2: 259–271.

[ece371886-bib-0040] McCauley, D. J. , M. L. Pinsky , S. R. Palumbi , J. A. Estes , F. H. Joyce , and R. R. Warner . 2015. “Marine Defaunation: Animal Loss in the Global Ocean.” Science 347: 1255641. 25593191 10.1126/science.1255641

[ece371886-bib-0041] McNab, B. K. 1963. “Bioenergetics and the Determination of Home Range Size.” American Naturalist 97: 133–140.

[ece371886-bib-0042] Minns, C. K. 1995. “Allometry of Home Range Size in Lake and River Fish.” Canadian Journal of Fisheries and Aquatic Sciences 52: 1499–1508.

[ece371886-bib-0043] Mitchell, M. S. , and R. A. Powell . 2004. “A Mechanistic Home Range Model for Optimal Use of Spatially Distributed Resources.” Ecological Modelling 177: 209–232.

[ece371886-bib-0044] Monti, F. , F. Ferretti , and N. Fattorini . 2024. “Intrinsic and Extrinsic Factors Modulating Vigilance and Foraging in Two Gregarious Foragers.” Behavioral Ecology 35, no. 1: arad114.

[ece371886-bib-0048] Pawar, S. , A. I. Dell , and V. M. Savage . 2012. “Dimensionality of Consumer Search Space Drives Trophic Interaction Strengths.” Nature 486: 485–489. 22722834 10.1038/nature11131

[ece371886-bib-0050] Peters, R. H. 1983. The Ecological Implications of Body Size. Cambridge University Press.

[ece371886-bib-0051] Pillans, R. D. , R. C. Babcock , D. P. Thomson , et al. 2017. “Habitat Effects on Home Range and Schooling Behaviour in a Herbivorous Fish (*Kyphosus bigibbus*) Revealed by Acoustic Tracking.” Marine and Freshwater Research 68: 1454–1467.

[ece371886-bib-0052] Pinsky, M. L. , A. M. Eikeset , D. J. McCauley , J. L. Payne , and J. M. Sunday . 2019. “Greater Vulnerability to Warming of Marine Versus Terrestrial Ectotherms.” Nature 569: 108–111. 31019302 10.1038/s41586-019-1132-4

[ece371886-bib-0054] Powell, R. A. 2000. “Animal Home Ranges and Territories and Home Range Estimators.” Research Techniques in Animal Ecology: Controversies and Consequences 442: 65–110.

[ece371886-bib-0057] Shokri, M. , F. Cozzoli , and A. Basset . 2025. “Metabolic Rate and Foraging Behaviour: A Mechanistic Link across Body Size and Temperature Gradients.” Oikos 2025, no. 1: e10817. 10.1111/oik.10817.

[ece371886-bib-0058] Shokri, M. , V. Marrocco , F. Cozzoli , F. Vignes , and A. Basset . 2024. “The Relative Importance of Metabolic Rate and Body Size to Space Use Behavior in Aquatic Invertebrates.” Ecology and Evolution 14, no. 5: e11253. 38770126 10.1002/ece3.11253 PMC11103644

[ece371886-bib-0075] Silva, I. , C. H. Fleming , M. J. Noonan , et al. 2021. “Autocorrelation‐Informed Home Range Estimation: A Review and Practical Guide.” 10.32942/osf.io/23wq7.

[ece371886-bib-0059] Tamburello, N. , I. M. Côté , and N. K. Dulvy . 2015. “Energy and the Scaling of Animal Space Use.” American Naturalist 186: 196–211. 10.1086/682070 26655149

[ece371886-bib-0060] Udyawer, V. , C. Huveneers , F. Jaine , et al. 2022. “Scaling of Activity Space in Marine Organisms Across Latitudinal Gradients.” American Naturalist 201, no. 4: 586–602. 10.1086/723405 36958006

[ece371886-bib-0067] van Beest, F. M. , I. M. Rivrud , L. E. Loe , J. M. Milner , and A. Mysterud . 2011. “What Determines Variation in Home Range Size Across Spatiotemporal Scales in a Large Browsing Herbivore?” Journal of Animal Ecology 80, no. 4: 771–785. 10.1111/j.1365-2656.2011.01825.x. 21388373

[ece371886-bib-0061] Vander Wal, E. , M. P. Laforge , and P. D. McLoughlin . 2014. “Density Dependence in Social Behaviour: Home Range Overlap and Density Interacts to Affect Conspecific Encounter Rates in a Gregarious Ungulate.” Behavioral Ecology and Sociobiology 68: 383–390.

[ece371886-bib-0066] Wang, M. , and V. Grimm . 2007. “Home Range Dynamics and Population Regulation: An Individual‐Based Model of the Common Shrew *Sorex araneus* .” Ecological Modelling 205, no. 3–4: 397–409.

[ece371886-bib-0062] West, G. B. , J. H. Brown , and B. J. Enquist . 1997. “A General Model for the Origin of Allometric Scaling Laws in Biology.” Science 276: 122–126. 9082983 10.1126/science.276.5309.122

[ece371886-bib-0064] Zuur, A. , and E. Ieno . 2016. “A Protocol for Conducting and Presenting Results of Regression‐Type Analyses.” Methods in Ecology and Evolution 7: 636–645.

[ece371886-bib-0065] Zuur, A. , E. N. Ieno , N. Walker , A. A. Saveliev , and G. M. Smith . 2009. Mixed Effects Models and Extensions in Ecology With R. Springer‐Verlag.

