# Supplementary material for: Behavioural Constraints to Home Range Allometries in Aquatic Organisms
Source: Ecol Evol. 2025 Jul 30;15(8):e71886. doi: 10.1002/ece3.71886 (PMC12311223; doi:10.1002/ece3.71886)
Supplement: Supplementary file 1 — Table S1. [file ECE3-15-e71886-s001.pdf]

OIKOS
ADVANCING ECOLOGY

Supplemental Information for:

Behavioural constraints to home range allometries: a meta-analysis on aquatic organisms

Table of Contents:

Table S1	Page 1

Table S1: Result of model selections conducted to investigate size adjusted home range (residual) variation in relation to behavioural traits in aquatic organisms. The top-ranked, non-nested models within ΔAICc<2 are shown in bold, with relevant degrees of freedom (df), logLik, AICc, ΔAICc and probability of being the best model (standardised weight). The first not-selected model (not bolded) is also shown for comparison.

**Response variable (n=161)**	**Food type**	**Migration**	**Sociability**	**Vertical distribution**	**Food type X Migration**	**Food type X Vertical distribution**	**Migration X Vertical distribution**	**Food type X Migration X Vertical distribution**	**df**	**logLik**	**AICc**	**ΔAICc**	**Weight**
Residuals of the hr~bs relationship (log 10)	x			x					**6**	**-184.713**	**382.0**	**0.00**	**0.197**
	x	x		x	x				**8**	**-183.053**	**383.1**	**1.08**	**0.115**
	x	x		x					**7**	**-184.222**	**383.2**	**1.20**	**0.108**
	x			x		x			7	-184.660	384.1	2.08	0.070
